# Supplementary material for: Food and water intake are regulated by distinct central amygdala circuits revealed using intersectional genetics
Source: Nat Commun. 2025 Mar 29;16:3072. doi: 10.1038/s41467-025-58144-3 (PMC11954953; doi:10.1038/s41467-025-58144-3)
Supplement: Supplementary file 1 — Supplementary information [file 41467_2025_58144_MOESM1_ESM.pdf]

Supplementary Information

Food and water intake are regulated by distinct central amygdala circuits revealed using intersectional genetics

Federica Fermani[1], Simon Chang[2], Ylenia Mastrodicasa[1], Christian Peters[1], Louise Gaitanos[1], Pilar L. Alcala Morales[1], Charu Ramakrishnan[3], Karl Deisseroth[3,4,5], Rüdiger Klein[1,6].

[1]Department of Molecules – Signaling – Development, Max-Planck Institute for Biological Intelligence, 82152 Martinsried, Germany.

[2] Cellular Neurobiology, Department of Behavioral and Molecular Neurobiology, University of Regensburg, Regensburg, Germany

[3] Department of Bioengineering, Stanford University, 94305 Stanford, USA

[4] Department of Psychiatry & Behavioral Sciences, Stanford University, 94305 Stanford, USA

[5] Howard Hughes Medical Institute, Stanford University, 94305 Stanford, USA

[6]Lead Contact

Correspondence: ruediger.klein@bi.mpg.de (R.K.)

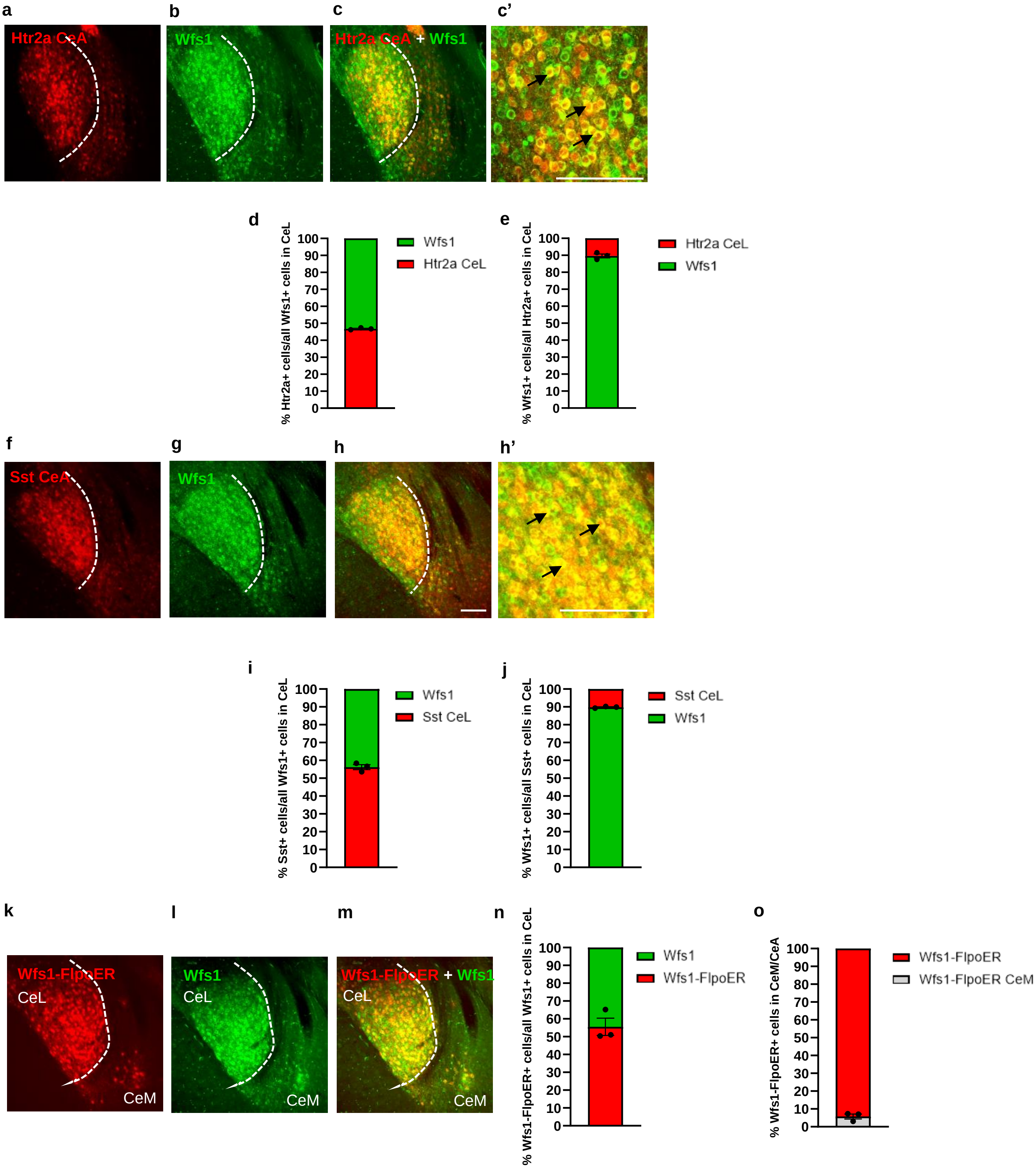

Supplementary Fig.1

Supplementary Figure 1 related to Figure 1. An intersectional strategy to dissect CeL and CeM subpopulations

a. tdTomato fluorescence in adult Htr2a-Cre;Ai9 mice. Stippled white line indicates the border between CeL and CeM.

b. Endogenous Wfs1 immunoreactivity in CeL.

c. Merge of panels a and b. **c'**. High magnification image of panel c. Black arrows highlight examples of cells double positive for both tdTom and Wfs1.

d. Fraction of Htr2a-Cre expressing neurons (46.8±0.3%) among all Wfs1-positive cells in CeL (n = 9 sections from 3 mice).

e. Fraction of Wfs1-positive neurons (89.6±1.2%) among all Htr2a-Cre expressing cells in CeL (n = 9 sections from 3 mice).

f. Expression of tdTomato in adult Sst-Cre;Ai9 mice.

g. Endogenous Wfs1 immunoreactivity in CeL.

h. Merge of panels f and g. **h'**. High magnification image of panel h. Black arrows highlight examples of cells colocalizing of both Sst and Wfs1.

i. Fraction of Sst-Cre-expressing cells (56.2±1.4%) among all Wfs1-positive cells in CeL (9 sections/3 mice).

j. Fraction of Wfs1-positive neurons (89.8±0.3%) among all Sst-Cre-expressing cells in CeL (9 sections/3 mice).

k-o. Validation of FlpoER expression. **k.** The activity of tamoxifen-inducible FlpoER in the CeA of Wfs1-FlpoER;FPDi mice leads to expression of mCherry in CeL.

l. Endogenous Wfs1 immunoreactivity in CeL.

m. Merge of panels k and l.

n. Fraction of cells expressing mCherry (transgenic Wfs1-FlpoER; 55.5±4.8%) among all Wfs1-positive cells in CeL (n=3 mice, 3 sections per brain).

o. Fraction of mCherry-positive cells in the CeM (5.8±1.4%) among all mCherry- positive cells in the CeA. (n=3 mice, 3 sections per brain). Values = Mean± SEM**.** Scale bar: 115 µm.

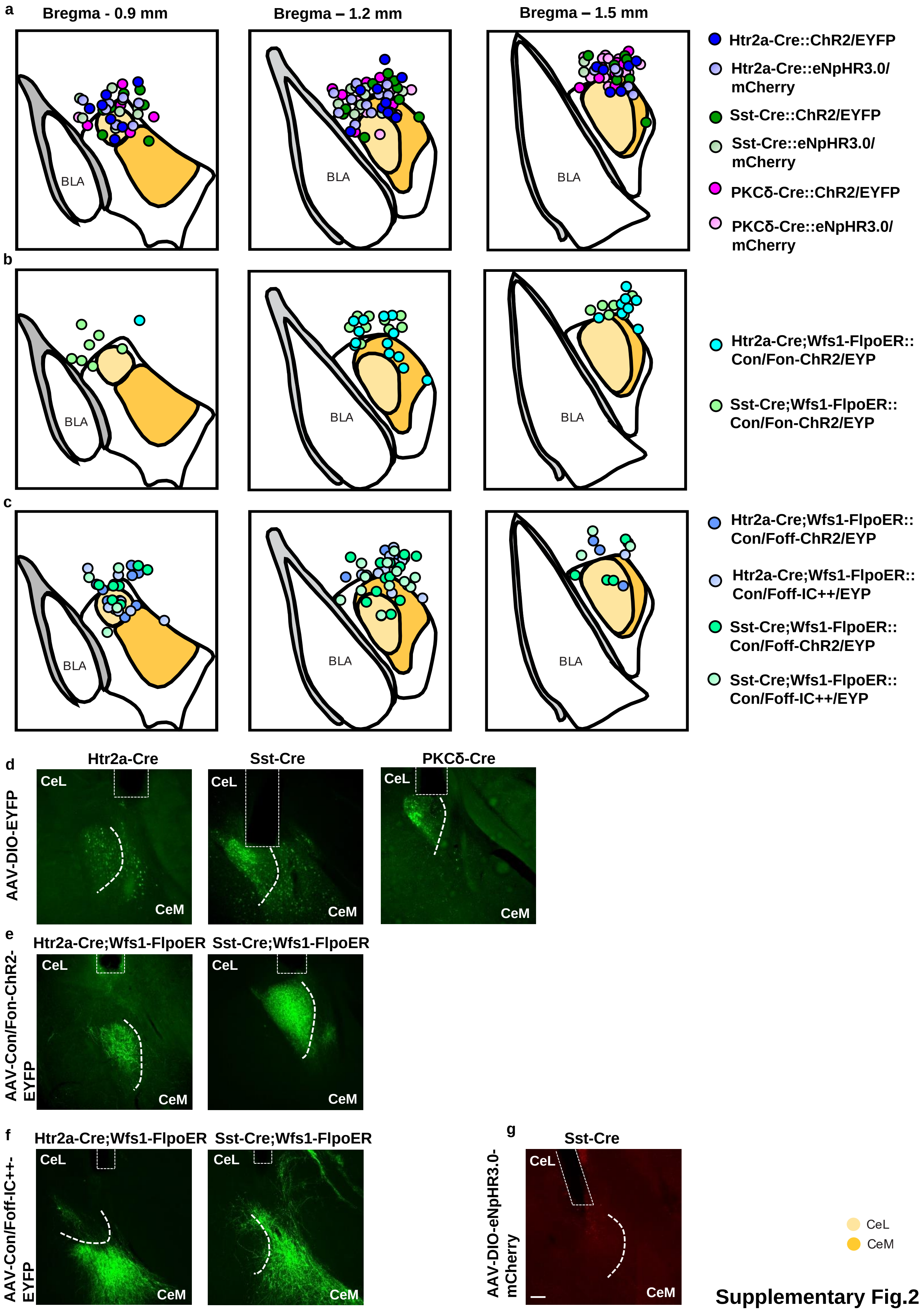

Supplementary Fig.2

Supplementary Figure 2 related to Figure 2 and Figure 3. Description of optical fiber placement

a. Diagram illustrating optic fiber placement in the CeA of mice injected with Cre-dependent viruses expressed throughout the CeA (Htr2a-Cre::ChR2/EYFP 22 out of 22 mice, Htr2a-Cre::eNpHR3.0/mCherry 22 out of 24 mice, PKCδ-Cre::ChR2/EYFP 19 out of 21 mice, PKCδ-Cre::eNpHR3.0/mCherry 24 out of 24 mice, Sst-Cre::ChR2/EYFP 26 out of 28 mice, Sst-Cre::eNpHR3.0/mCherry 24 out of 28 mice).

b. Diagram illustrating optic fiber placement in the CeA of mice injected with Con/Fon viruses expressed throughout the CeL (Htr2a-Cre;Wfs1-FlpoER::Con/Fon-ChR2/EYFP 22 out of 22 mice, Sst-Cre;Wfs1-FlpoER::Con/Fon-ChR2/EYFP 22 out of 22 mice).

c. Diagram illustrating optic fiber placement in the CeA of mice injected with Con/Foff viruses expressed throughout the CeM (Htr2a-Cre;Wfs1-FlpoER::Con/Foff-ChR2/EYFP 36 out of 40 mice, Htr2a-Cre;Wfs1-FlpoER::Con/Foff-IC++/EYFP 21 out of 21 mice, Sst-Cre;Wfs1-FlpoER::Con/Foff-ChR2/EYFP 41 out of 43 mice, Sst-Cre;Wfs1-FlpoER::Con/Foff-IC++/EYFP 19 out of 19 mice).

d. Representative pictures showing the optic fiber placement in the CeA of Htr2a-Cre, Sst-Cre and PKCδ-Cre mice injected with AAV-DIO-EYFP virus.

e. Representative pictures showing the optic fiber placement in the CeA of Htr2a-Cre;Wfs1-FlpoER and Sst-Cre;Wfs1-FlpoER mice injected with AAV-Con/Fon-ChR2-EYFP virus.

f. Representative pictures showing the optic fiber placement in the CeA of Htr2a-Cre;Wfs1-FlpoER and Sst-Cre;Wfs1-FlpoER mice injected with AAV-Con/Foff-IC++-EYFP virus.

g. Representative picture of a mouse excluded from the behavioral analysis due to lack of viral expression in the CeA.

Scale bar: 126 μm.

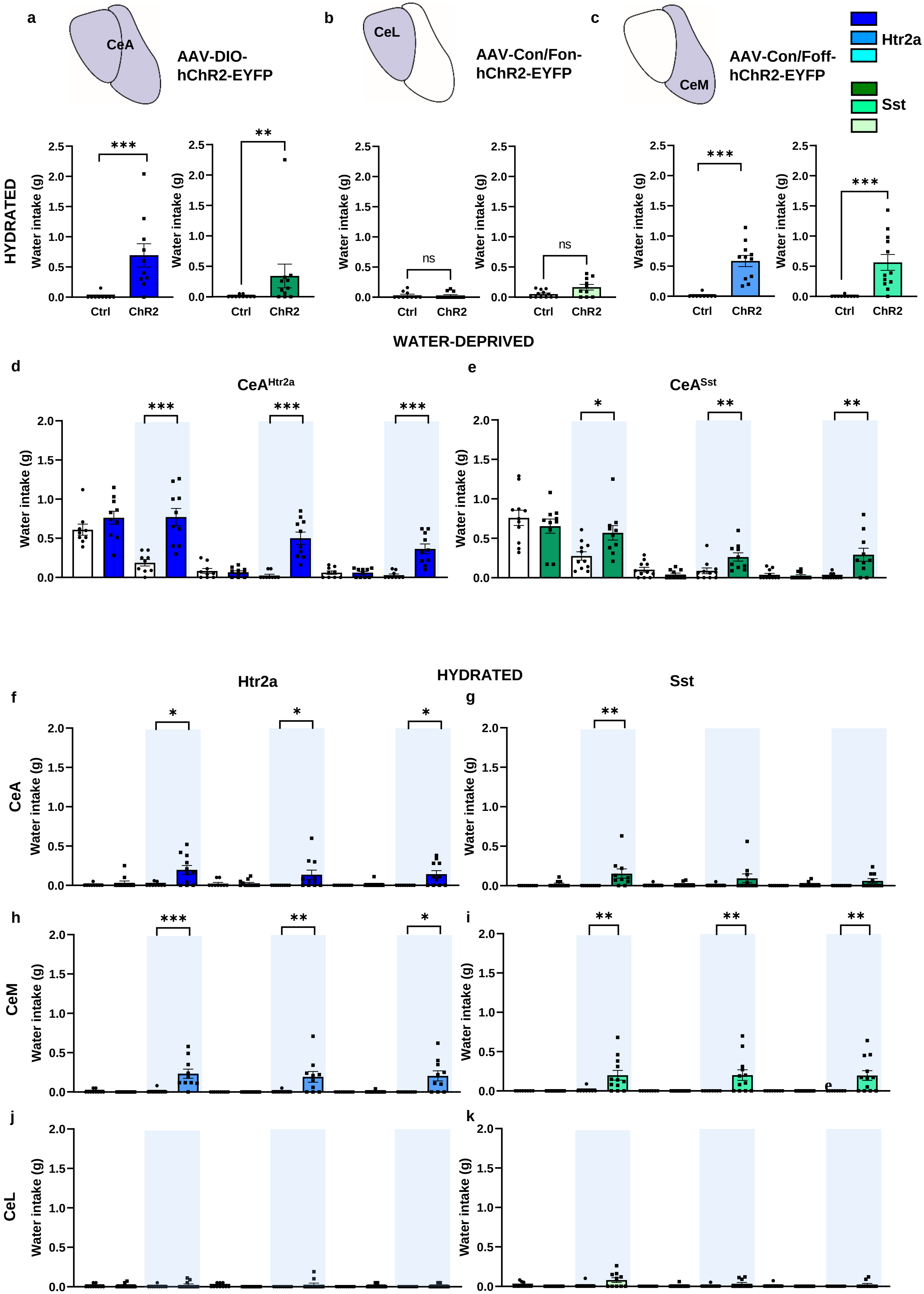

Supplementary Fig.3

Supplementary Figure 3 related to Figure 2: Water consumption induced by photoactivation of different subpopulations of Htr2a-Cre and Sst-Cre expressing neurons

a-c. Water intake during 30 min by normally hydrated mice during photoactivation of (**a**) the entire CeA populations of Htr2a-Cre (blue) or Sst-Cre expressing neurons (green), (**b**) the respective CeL subpopulations, or (**c**) the respective CeM subpopulations, compared to photoactivated control animals (CeA^{Htr2a}: Mann-Whitney two-tailed U test p=0.0002, U=5; CeA^{Sst}: Mann-Whitney two-tailed U test p=0.0038, U=18; CeL^{Htr2a}: Mann-Whitney two-tailed U test p=0.8939, U=58; CeL^{Sst}: Mann-Whitney two-tailed U test p=0.0699, U=33; CeM^{Htr2a}: Mann-Whitney two-tailed U test p<0.0001, U=0; CeM^{Sst}: Mann-Whitney two-tailed U test p<0.0001, U=5.500). (CeA^{Htr2a}: n=9 Ctrl, n=10 ChR2. CeA^{Sst}: n=10 Ctrl, n=11 ChR2. CeL^{Htr2a}: n=10 Ctrl, n=12 ChR2. CeL^{Sst}: n=12 Ctrl, n=10 ChR2. CeM^{Htr2a}: n=9 Ctrl, n=11 ChR2. CeM^{Sst}: n=10 Ctrl, n=12 ChR2 mice).

d,e. Water intake during a 10-minute Light OFF/ON behavioral paradigm by water-deprived mice with photoactivated CeA^{Htr2a} (blue) or CeA^{Sst} neurons (green), compared to controls (Htr2a: 10-20 min: unpaired two-tailed t test p=0.0002, t=4.729. 30-40 min: Mann-Whitney two-tailed U test p<0.0001, U=0. 50-60 min: Mann-Whitney two-tailed U test p<0.0001, U=1.500. Sst: 10-20 min: unpaired two-tailed t test p=0.0103, t=2.847. 30-40 min: Mann-Whitney two-tailed U test p=0.0038, U=15.50. 50-60 min: Mann-Whitney two-tailed U test p=0.0014, U=14). (CeA^{Htr2a}: n=9 Ctrl, n=10 ChR2. CeA^{Sst}: n=11 Ctrl, n=10 ChR2 mice).

f-k. The same 10-minute Light OFF/ON paradigm was repeated with hydrated animals.

f,g. Photoactivation of the entire populations of CeA^{Htr2a} or CeA^{Sst} neurons promoted drinking (Htr2a: 10-20 min: Mann-Whitney two-tailed U test p=0.0107, U=18.50. 30-40 min: two-tailed Wilcoxon signed rank test p=0.0156. 50-60 min: two-tailed Wilcoxon signed rank test p=0.0156. Sst: 10-20 min: two-tailed Wilcoxon signed rank test p=0.0078). (CeA^{Htr2a}: n=9 Ctrl, n=11 ChR2. CeA^{Sst}: n=10 Ctrl, n=10 ChR2 mice).

h,i. Photoactivation of the CeM^{Htr2a} or CeM^{Sst} subpopulations was sufficient to induce water uptake (Htr2a: 10-20 min Mann-Whitney two-tailed U test p=0.0001, U=5.500. 30-40 min: Mann-Whitney two-tailed U test p=0.0031, U=16.50. 50-60 min: two-tailed Wilcoxon signed rank test p=0.0156. Sst: 10-20 min Mann-Whitney two-tailed U test p=0.0067, U=15.50. 30-40 min: two-tailed Wilcoxon signed rank test p=0.0078. 50-60 min: two-tailed Wilcoxon signed rank test p=0.0078). (CeM^{Htr2a}: n=10 Ctrl, n=10 ChR2. CeM^{Sst}: n=8 Ctrl, n=12 ChR2 mice).

j,k. No effect was observed after photoactivation of the CeL^{Htr2a} or CeL^{Sst} subpopulations (Htr2a: 10-20 min Mann-Whitney two-tailed U test p=0.3935, U=44.50. 30-40 min: two-tailed Wilcoxon signed rank test p=0.5000. 50-60 min: two-tailed Wilcoxon signed rank test p=0.5000. Sst: 10-20 min two-tailed Wilcoxon

signed rank test *p*=0.0625. 30-40 min: Mann-Whitney two-tailed U test *p*=0.1485, U=49.50-60 min: two-tailed Wilcoxon signed rank test *p*=0.5000). (CeL^{Htr2a}: n=10 Ctrl, n=11 ChR2. CeL^{Sst}: n=13 Ctrl, n=10 ChR2 mice). Value = Mean ± SEM.

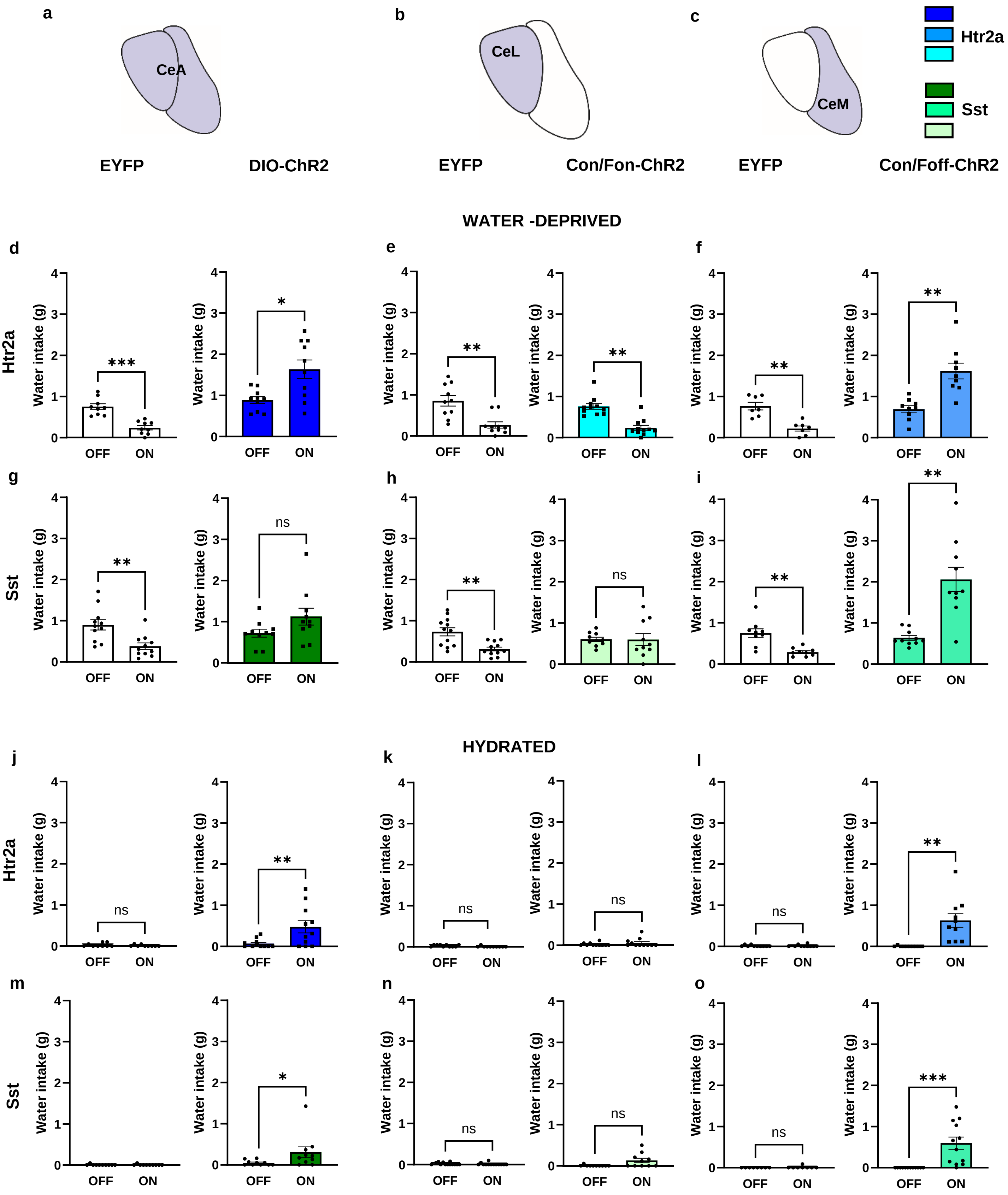

Supplementary Fig.4

Supplementary Figure 4 related to Figure 2: Comparisons of water consumption between photostimulated and unstimulated ChR2-expressing mice and controls

a-c. Schemes of CeA subregional expression of INTRSECT viruses.

d-i. Total amount of water consumed by water-deprived animals during a 10-min light-OFF epoch followed by a 10-min light-ON epoch.

d. Photostimulation of the entire population of CeA^{Htr2a} cells promoted water consumption compared to Light OFF periods. The opposite effect was observed in CeA^{Htr2a} controls (Htr2a::EYFP: paired two-tailed t test *p*=0.0006, t=5.434. Htr2a::ChR2: paired two-tailed t test *p*=0.0143, t=3.028.). (CeA^{Htr2a}: n=9 Ctrl, n=10 ChR2 mice).

e. No positive effect on water uptake of photostimulating CeL^{Htr2a} or controls (Htr2a::EYFP: two-tailed Wilcoxon matched-pairs signed rank test *p*=0.0059. Htr2a::ChR2: : two-tailed Wilcoxon matched-pairs signed rank test *p*=0.0029). (CeL^{Htr2a}: n=10 Ctrl, n=11 ChR2 mice).

f. Photostimulation of CeM^{Htr2a} cells increased water uptake compared to light OFF periods. No positive effect of the light in CeM^{Htr2a} controls (Htr2a::EYFP: paired two-tailed t test *p*=0.0037, t=4.602. Htr2a::ChR2: paired two-tailed t test *p*=0.0059, t=3.720). (CeM^{Htr2a}: n=7 Ctrl, n=9 ChR2 mice).

g. Photostimulation of the entire population of CeA^{Sst} cells did not promote water consumption compared to Light OFF periods. No positive effect of the light in CeA^{Sst} controls (Sst::EYFP: paired two-tailed t test *p*=0.0012, t=4.486. Sst::ChR2: paired two-tailed t test *p*=0.1054, t=1.800.). (CeA^{Sst}: n=11 Ctrl, n=10 ChR2 mice).

h. No positive effect on water uptake of photostimulating CeL^{Sst} or controls (Sst::EYFP: paired two-tailed t test *p*=0.0018, t=4.079. Sst::ChR2: paired two-tailed t test *p*=0.9805, t=0.02512). (CeL^{Sst}: n=12 Ctrl, n=10 ChR2 mice).

i. Photostimulation of CeM^{Sst} cells increased water uptake compared to light OFF periods. No positive effect of the light in CeM^{Sst} controls (Sst::EYFP: paired two-tailed t test *p*=0.0017, t=4.625. Sst::ChR2: paired two-tailed t test *p*=0.0014, t=4.568). (CeM^{Sst}: n=9 Ctrl, n=10 ChR2 mice).

j-o. Total water intake by hydrated mice during the 10-minutes Light ON/OFF paradigm.

j. Photostimulation of the entire population of CeA^{Htr2a} cells promoted water consumption compared to Light OFF periods. No positive effect of the light in CeA^{Htr2a} controls (Htr2a::EYFP: two-tailed Wilcoxon matched-pairs signed rank test *p*=0.6250. Htr2a::ChR2: two-tailed Wilcoxon matched-pairs signed rank test *p*=0.0078). (CeA^{Htr2a}: n=9 Ctrl, n=11 ChR2 mice).

k. No positive effect on water uptake of photostimulating CeL^{Htr2a} cells or controls (Htr2a::EYFP: two-tailed Wilcoxon matched-pairs signed rank test *p*=0.1250. Htr2a::ChR2: : two-tailed Wilcoxon matched-pairs signed rank test *p*=0.1250). (CeL^{Htr2a}: n=10 Ctrl, n=11 ChR2 mice).

l. Photostimulation of CeM^{Htr2a} cells increased water uptake compared to light OFF periods. No positive effect of the light in CeM^{Htr2a} controls (Htr2a::EYFP: two-tailed Wilcoxon matched-pairs signed rank test *p*>0.9999. Htr2a::ChR2: : two-tailed Wilcoxon matched-pairs signed rank test *p*=0.0020). (CeM^{Htr2a}: n=10 Ctrl, n=10 ChR2 mice).

m. Photostimulation of the entire population of CeA^{Sst} cells did not promote water consumption compared to Light OFF periods. No positive effect of the light in CeA^{Sst} controls (Sst::EYFP: two-tailed Wilcoxon matched-pairs signed rank test *p*>0.9999. Sst::ChR2: two-tailed Wilcoxon matched-pairs signed rank test *p*=0.0391). (CeA^{Sst}: n=10 Ctrl, n=10 ChR2 mice).

n. No positive effect on water uptake of photostimulating CeL^{Sst} cells or controls (Sst::EYFP: two-tailed Wilcoxon matched-pairs signed rank test *p*=0.5000. Sst::ChR2: two-tailed Wilcoxon matched-pairs signed rank test *p*=0.0625). (CeL^{Sst}: n=13 Ctrl, n=10 ChR2 mice).

o. Photostimulation of CeM^{Sst} cells increased water uptake compared to light OFF periods. No positive effect of the light in CeM^{Sst} controls (Sst::EYFP: two-tailed Wilcoxon matched-pairs signed rank test *p*>0.9999. Sst::ChR2: two-tailed Wilcoxon matched-pairs signed rank test *p*=0.0010). (CeM^{Sst}: n=8 Ctrl, n=12 ChR2 mice). Value = Mean ± SEM.

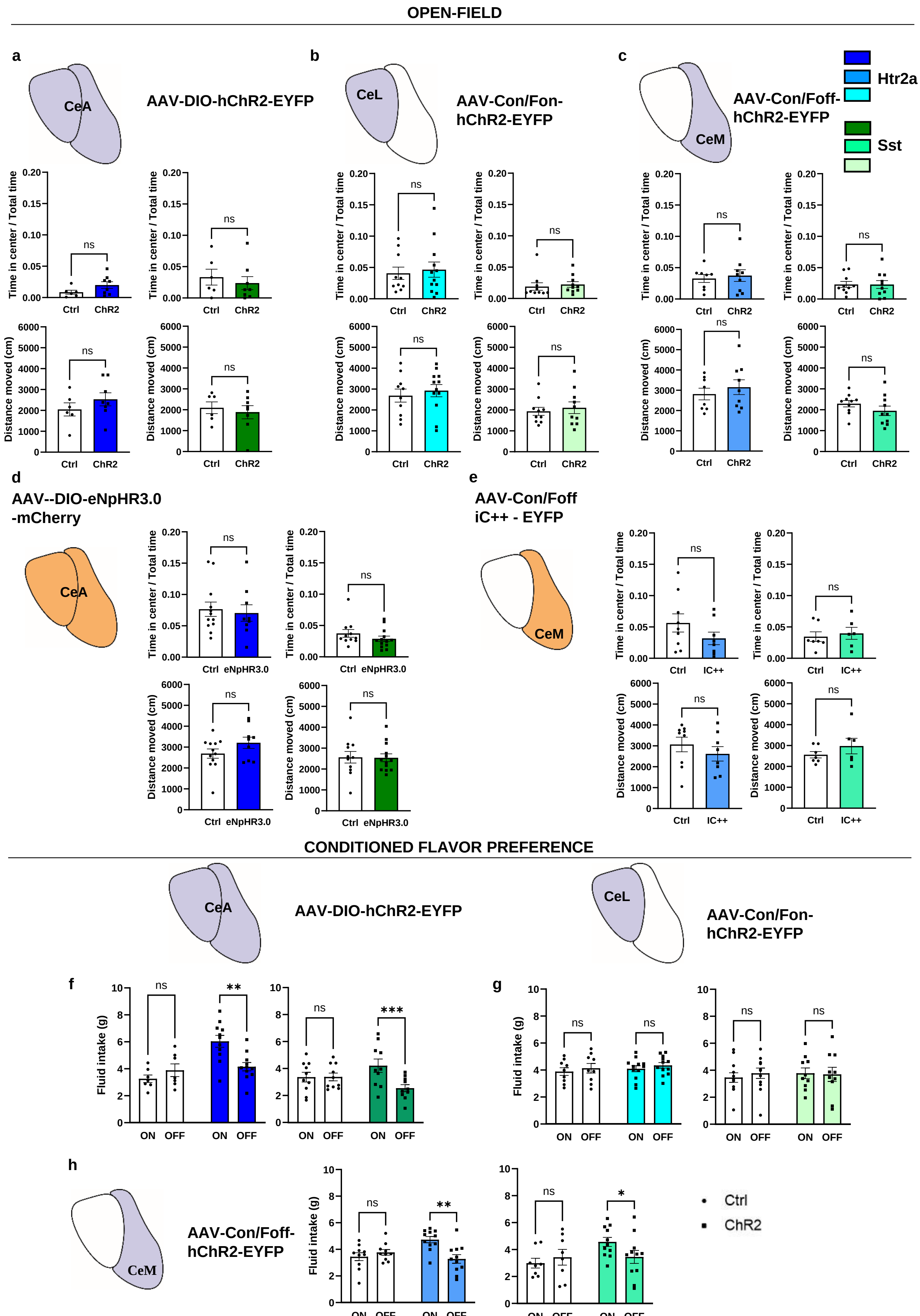

Supplementary Fig.5

Supplementary Figure 5 related to Figure 5: Effects of optogenetic manipulation of Htr2a and Sst subpopulations in open field and conditioned flavor preference task

a. Portion of time in the center-zone and total distance travelled during an open-field behavioral test (OF) of mice in which the entire populations of CeA^{Htr2a} (blue) or CeA^{Sst} (green) neurons were photostimulated in comparison to control mice (Time: CeA^{Htr2a}: unpaired two-tailed t test *p*=0.1222, t=1.663. CeA^{Sst}: Mann-Whitney two-tailed U test *p*=0.5987, U=19.50. Distance: CeA^{Htr2a}: unpaired two tailed t test *p*=0.3075, t=1.066. CeA^{Sst}: unpaired two tailed t test *p*=0.6318, t=0.4917). (CeA^{Htr2a} and CeA^{Sst}: n=6 Ctrl, n=8 ChR2 mice).

b. Same as panel a except for CeL^{Htr2a} (blue) or CeL^{Sst} (green) neurons (Time: CeL^{Htr2a}: Mann-Whitney two-tailed U test *p*=0.7713, U=55. CeL^{Sst}: Mann-Whitney two-tailed U test *p*=0.2245, U=33.50 Distance: CeL^{Htr2a}: unpaired two-tailed t test *p*=0.5855, t=0.5543. CeL^{Sst}: unpaired two-tailed t test *p*=0.6180, t=0.5075.). (CeL^{Htr2a}: n=10 Ctrl, n=12 ChR2. CeL^{Sst}: n=10 Ctrl, n=10 ChR2 mice).

c. Same as panel a except for CeM^{Htr2a} (blue) or CeM^{Sst} (green) neurons (Time: CeM^{Htr2a}: unpaired two-tailed t test *p*=0.6934, t=0.4019. CeM^{Sst}: unpaired two-tailed t test *p*=0.9833, t=0.02122. Distance: CeM^{Htr2a}: unpaired two-tailed t test *p*=0.4852, t=0.7157. CeM^{Sst}: unpaired two-tailed t test *p*=0.2274, t=1.250). (CeM^{Htr2a}: n=8 Ctrl, n=9 ChR2. CeM^{Sst}: n= 10 Ctrl, n= 10 ChR2 mice).

d. Same as panel a except for CeA^{Htr2a} (blue) or CeA^{Sst} (green) neurons while being photoinhibited (Time: CeA^{Htr2a}: Mann-Whitney two-tailed U test *p*=0.8480, U=51. CeA^{Sst}: Mann-Whitney two-tailed U test *p*=0.1500, U=46. Distance: CeA^{Htr2a}: unpaired two-tailed t test *p*=0.1515, t=1.494. CeA^{Sst}: unpaired two-tailed t test *p*=0.9373, t=0.07962). (CeA^{Htr2a}: n=12 Ctrl, n=9 eNpHR3.0. CeA^{Sst}: n=11 Ctrl, n=13 eNpHR3.0 mice).

e. Same as panel a except for CeM^{Htr2a} (blue) or CeM^{Sst} (green) neurons while being photoinhibited (Time: CeM^{Htr2a}: unpaired two-tailed t test *p*=0.1935, t=1.361. CeM^{Sst}: unpaired two-tailed t test *p*=0.6783, t=0.4260. Distance: CeM^{Htr2a}: Mann-Whitney two-tailed U test *p*=0.3704, U=26. CeM^{Sst}: unpaired two-tailed t test *p*=0.3020, t=1.083). (CeM^{Htr2a}: n= 9 Ctrl, n= 8 IC++. CeM^{Sst}: n= 7 Ctrl, n= 6 IC++ mice)

f. Fluid intake during conditioned flavor preference task by mice in which the entire populations of CeA^{Htr2a} (blue) or CeA^{Sst} (green) neurons were photostimulated in comparison to unstimulated mice (Htr2a: main effect ChR2: Two-way ANOVA, $F(1,16)$ =11.94, *p*=0.0033; Bonferroni post-hoc test *p*=0.0014. Sst: main effect Light, Two-way ANOVA, $F(1,18)$ =10.30, *p*=0.0049; Bonferroni post-hoc test *p*=0.0005). (CeA^{Htr2a}: n=7 Ctrl, n=11 ChR2. CeA^{Sst}: n=10 Ctrl, n=10 ChR2 mice).

g. Same as panel f except for CeL^{Htr2a} (blue) or CeL^{Sst} (green) neurons (Htr2a: main effect ChR2: Two-way ANOVA, $F(1,19)$ =0.4309, *p*=0.5194.

Sst: main effect ChR2: Two-way ANOVA, $F(1,20) = 0.04635$, $p = 0.8317$). (CeL^{Htr2a}: n=9 Ctrl, n=12 ChR2. CeL^{Sst}: n=12 Ctrl, n=10 ChR2 mice).

h. Same as panel f except for CeM^{Htr2a} (blue) or CeM^{Sst} (green) neurons (Htr2a: main effect LightxChR2, Two-way ANOVA, $F(1,19) = 11.44$, $p = 0.0031$; Bonferroni post-hoc test $p = 0.0015$. Sst: main effect LightxChR2, Two-way ANOVA, $F(1,17) = 4.966$, $p = 0.0396$; Bonferroni post-hoc test $p = 0.0499$). (CeM^{Htr2a}: n=10 Ctrl, n=11 ChR2. CeM^{Sst}: n=8 Ctrl, n=11 ChR2 mice). Value = Mean ± SEM.

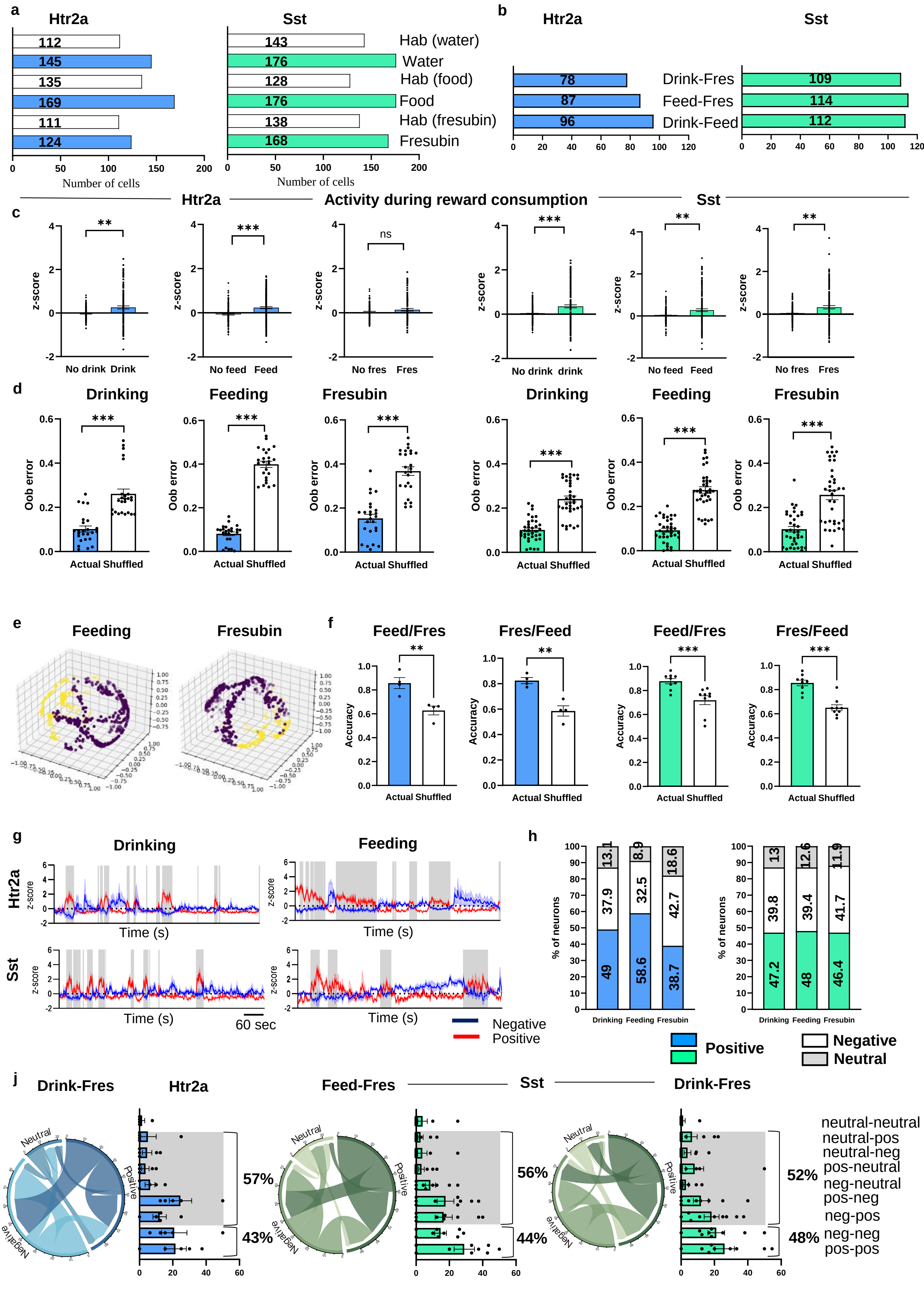

Supplementary Fig.6

Supplementary Figure 6 related to Figure 6. Calcium responses of CeM^{Htr2a} and CeM^{Sst} neurons to different rewarding stimuli

a. Total numbers of recorded CeM^{Htr2a} and CeM^{Sst} cells in the 10-min habituation and the 10-min reward exposure (water, food or fresubin).

b. Numbers of CeM^{Htr2a} and CeM^{Sst} neurons longitudinally detected during drinking-feeding, feeding-Fresubin or drinking-Fresubin behavioral paradigms.

c. Average z-score comparisons between periods of inactivity and bouts of reward consumption of CeM^{Htr2a} and CeM^{Sst} neurons (two-tailed Wilcoxon matched-pairs signed rank test. CeM^{Htr2a}: drinking, *p*=0.0079, n=145; feeding, *p*<0.0001, n=169; Fresubin, *p*=0.4580, n=124. CeM^{Sst}: drinking, *p*=0.0008, n=176; feeding, *p*=0.0052, n=176; Fresubin, *p*=0.0065, n=168).

d. Out of bag error of Random Forest for Htr2a and Sst embeddings (Htr2a Drinking, Feeding, Fresubin: two-tailed Wilcoxon matched-pairs signed rank test *p*<0.0001. Sst Drinking, Feeding, Fresubin: two-tailed Wilcoxon matched-pairs signed rank test *p*<0.0001). (Out of bag error of behavior compared to shuffled data. Htr2a drinking,feeding,fresubin n=25; Sst drinking,feeding,fresubin n=36).

e. Representative images of CEBRA generated embeddings of Htr2a neurons longitudinally detected in feeding-Fresubin.

f. Accuracy of behavioral decoding through Random Forest, employing CEBRA embeddings of Htr2a (in blue) and Sst (in green) neurons longitudinally detected during feeding and Fresubin consumption. The embeddings derived from feeding behavior are used for predicting Fresubin and vice versa (Htr2a: Feed/Fres: paired two-tailed t test *p*=0.0042, t=7.891. Fres/Feed: paired two-tailed t test *p*=0.0043, t=7.838. Sst: Feed/Fres: paired two-tailed t test *p*=0.0002, t=6.559. Fres/Feed: paired two-tailed t test *p*=0.0002, t=6.448). (Prediction scores of accuracy of behavior compared to shuffled data. Htr2a: Actual Shuffled: n=4. Sst: Actual-Shuffled: n=9).

g. Representative average activity traces of CeM^{Htr2a} and CeM^{Sst} neurons recorded from one mouse each during 10-minute exposure to water or food. In red the positive and in blue the negative correlated cells. In grey the consummatory bouts.

h. Graphs showing the percentages of neurons positively or negatively correlated (blue for CeM^{Htr2a}, green for CeM^{Sst}) to drinking, feeding and Fresubin consumption.

j. Chord diagrams and bar graphs depicting stable or unstable correlations of the same CeM^{Htr2a} and CeM^{Sst} neurons from drinking to Fresubin and from feeding to Fresubin. Data from neurons with unstable correlations highlighted in gray (Htr2a drinking-Fresubin stable v.s. unstable: Mann-Whitney two-tailed U test *p*=0.1587, U=4.500. Sst feeding-Fresubin stable v.s. unstable: unpaired two-tailed t test *p*=0.3176,

t=1.032. Sst drinking-Fresubin stable v.s. unstable: unpaired two-tailed t test p=0.6533, t=0.4577). (Htr2a: n= 5, Sst: n=9 mice). Value = Mean ± SEM.

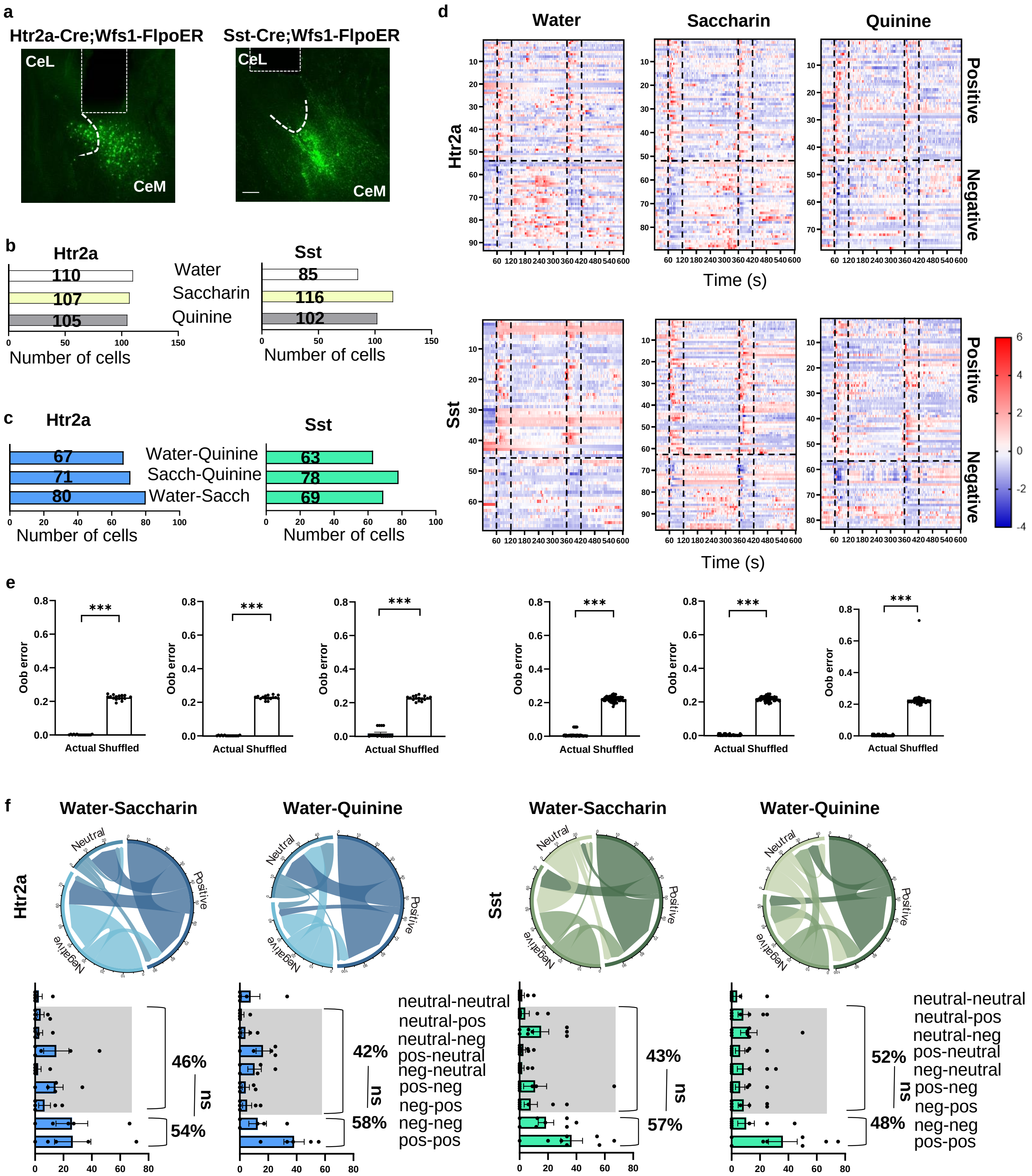

Supplementary Fig.7

Supplementary Figure 7 related to Figure 7. Calcium responses of CeM^{Htr2a} and CeM^{Sst} cells to different salient stimuli

a. Representative pictures showing the optic fiber placement in the CeA of Htr2a-Cre;Wfs1-FlpoER and Sst-Cre;Wfs1-FlpoER mice injected with AAV-Con/Foff-GCaMP6m virus in the CeM. Scale bar: 126 µm.

b. Total numbers of CeM^{Htr2a} and CeM^{Sst} cells recorded during the 10-min water, saccharin and quinine sessions.

c. Numbers of CeM^{Htr2a} and CeM^{Sst} neurons longitudinally detected during water-saccharin, saccharin-quinine and water-quinine sessions.

d. Heatmap visualization of the activities of CeM^{Htr2a} and CeM^{Sst} neurons having positive or negative correlations with water, saccharin and quinine.

e. Out of bag error of Random Forest for Htr2a and Sst embeddings (Htr2a water,saccharin,quinine: two-tailed Wilcoxon matched-pairs signed rank test $p<0.0001$. Sst water,saccharin,quinine: Wilcoxon matched-pairs signed rank test $p<0.0001$). (Out of bag error of behavior compared to shuffled data. Htr2a water, saccharin, quinine n=16; Sst water, saccharin, quinine n=57).

f. Chord diagrams and bar graphs depicting the stable ("generalizers") or unstable correlation ("specializers") of the same CeM^{Htr2a} and CeM^{Sst} neurons when switching from water to saccharin and from water to quinine. Data from specializers highlighted in gray (Htr2a: water-saccharin generalizer v.s. specializers: unpaired two-tailed t test $p=0.4554$, t=0.7844. Water-quinine generalizer v.s. specializers: unpaired two-tailed t test $p=0.3546$, t=0.9826. Sst: water-saccharin generalizer v.s. specializers: Mann-Whitney two-tailed U test $p=0.0859$, U=15. Water-quinine generalizer v.s. specializers: unpaired two-tailed t test $p=0.7155$, t=0.3719). (Htr2a: n= 5, Sst: n=8 mice). Value = Mean ± SEM.

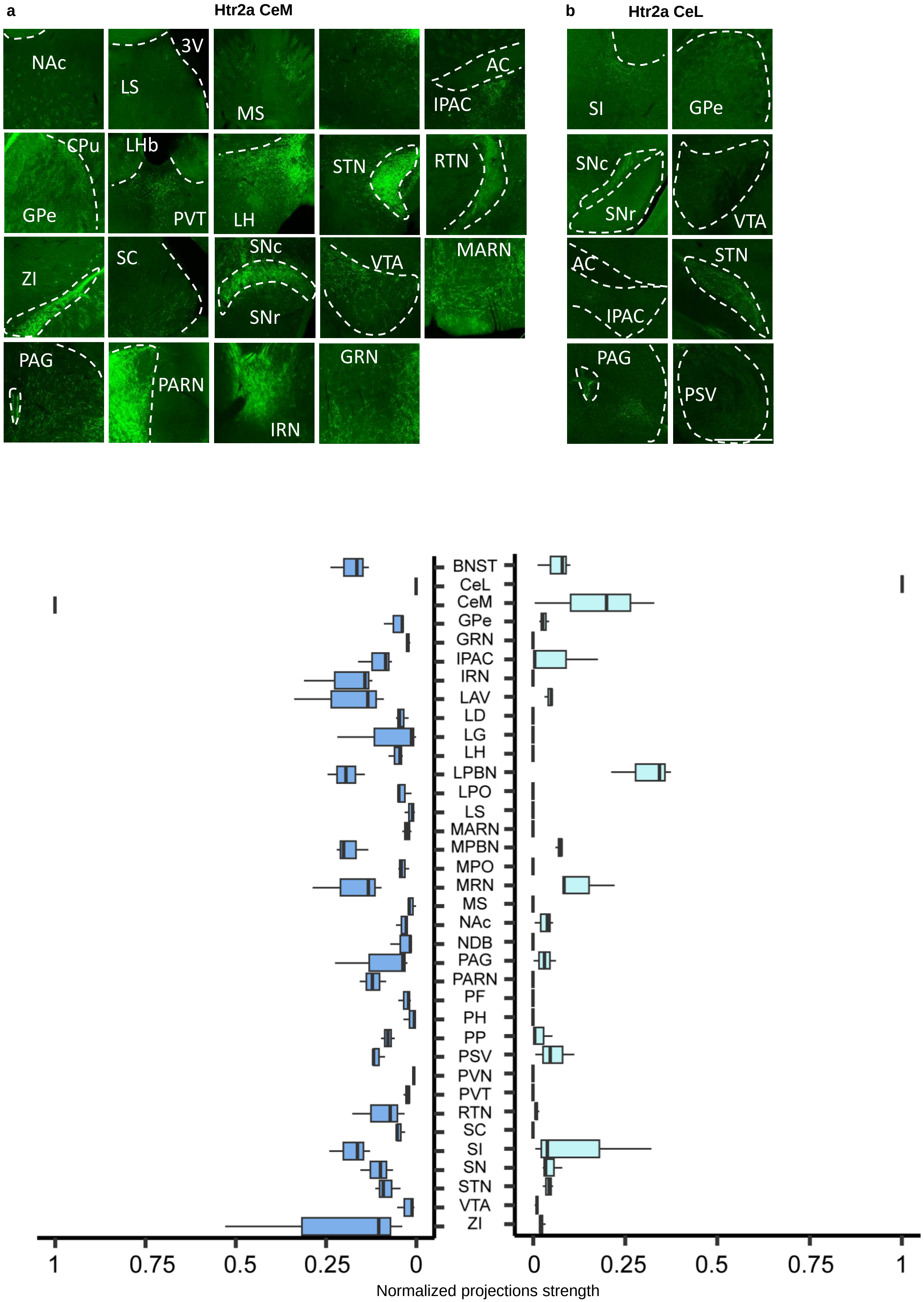

Supplementary Fig.8

Supplementary Figure 8 related to Figure 9. Long range projections of CeM and CeL Htr2a neurons

a. Images displaying brain areas receiving projections from CeM^{Htr2a} neurons and relative box plots of the quantifications (left side).

b. Images displaying brain areas receiving projections from CeL^{Htr2a} neurons and relative blox plot of the quantifications (right side). In these experiments Htr2a-Cre;Wfs1-FlpoER mice were injected either with a Con/Foff-EYFP or with a Con/Fon-EYFP virus. (n=3 mice per condition). Values= Median (Center) ±Min/Max (whiskers). 25 to 75 percentile (box). Scale bar: 500 µm.

Abbreviations: AC: anterior commissure. BNST: bed nucleus of the stria terminalis. CeL: central amygdala lateral part. CeM: central amygdala medial part. CPu: caudoputamen. GPe: globus pallidus, external segment. GRN: gigantocellular reticular nucleus. IPAC: interstitial nucleus of the anterior commissure. IRN: intermediate reticular nucleus. LAV: lateral vestibular nucleus. LD: lateral dorsal nucleus of the thalamus. LG: lateral geniculate complex. LH: lateral hypothalamus. LHb: lateral habenula. L/MPBN: lateral/medial parabrachial nucleus. LPO: lateral preoptic area. LS: lateral septal nucleus. MARN: magnocellular reticular nucleus. MPO: medial preoptic area. MRN: midbrain reticular. MS: medial septal nucleus. NAc: nucleus accumbens. NDB: diagonal band nucleus. PAG: periaqueductal gray. PARN: parvicellular reticular nucleus. PF: parafascicular nucleus. PH: posterior hypothalamic nucleus. PP: peripeduncular nucleus. PSV: principal sensory nucleus of the trigeminal. PVN: paraventricular nucleus of the hypothalamus. PVT: paraventricular nucleus of the thalamus. RTN: reticular thalamic nucleus. SC: superior colliculus. SI: Substantia innominata. SN: substantia nigra. STN: subthalamic nucleus. VTA: ventral tegmental area. ZI: zona incerta. 3V: third ventricle.

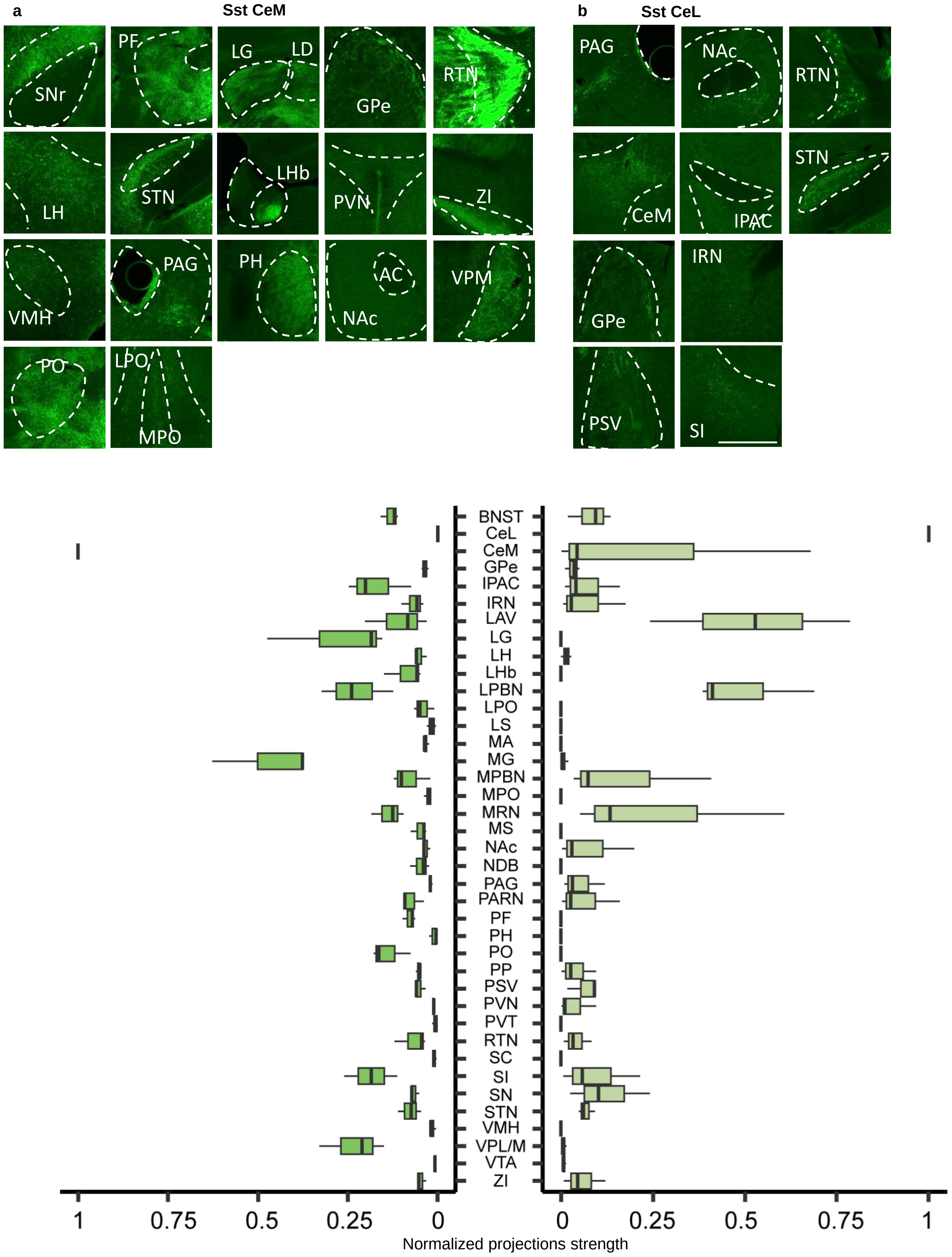

Supplementary Fig.9

Supplementary Figure 9 related to Figure 9. Long range projections of CeM and CeL Sst neurons

a. Images displaying brain areas receiving projections from CeM^{Sst} neurons and relative blox plot of the quantifications (left side).

b. Images displaying brain areas receiving projections from CeL^{Sst} neurons and relative blox plot of the quantifications (right side). In these experiments Sst-Cre;Wfs1-FlpoER mice were injected either with a Con/Foff-EYFP or with a Con/Fon-EYFP virus. (n=3 mice per condition). Values= Median (Center) ±Min/Max (whiskers). 25 to 75 percentile (box). Scale bar: 500 µm.

Abbreviations: AC: anterior commissure. BNST: bed nucleus of the stria terminalis. CeL: central amygdala lateral part. CeM: central amygdala medial part. GPe: globus pallidus, external segment IPAC: interstitial nucleus of the anterior commissure. IRN: intermediate reticular nucleus. LAV: lateral vestibular nucleus. LD: lateral dorsal nucleus of the thalamus. LG: lateral geniculate complex. LH: lateral hypothalamus. LHb: lateral habenula. L/mPBN: lateral/medial parabrachial nucleus. LPO: lateral preoptic area. LS: lateral septal nucleus. MA: magnocellular nucleus. MG: medial geniculate complex. MPO: medial preoptic area. MRN: midbrain reticular. MS: medial septal nucleus. NAc: nucleus accumbens. NDB: diagonal band nucleus. PAG: periaqueductal gray. PARN: parvicellular reticular nucleus. PF: parafascicular nucleus. PH: posterior hypothalamic nucleus. PO: posterior complex of the thalamus. PP: peripeduncular nucleus. PSV: principal sensory nucleus of the trigeminal. PVN: paraventricular nucleus of the hypothalamus. PVT: paraventricular nucleus of the thalamus. RTN: reticular thalamic nucleus. SC: superior colliculus. SI: Substantia innominata. SN: substantia nigra. STN: subthalamic nucleus. VMH: ventromedial hypothalamic nucleus. VPL/M: ventral posterolateral/medial nucleus of the thalamus. VTA: ventral tegmental area. ZI: zona incerta.

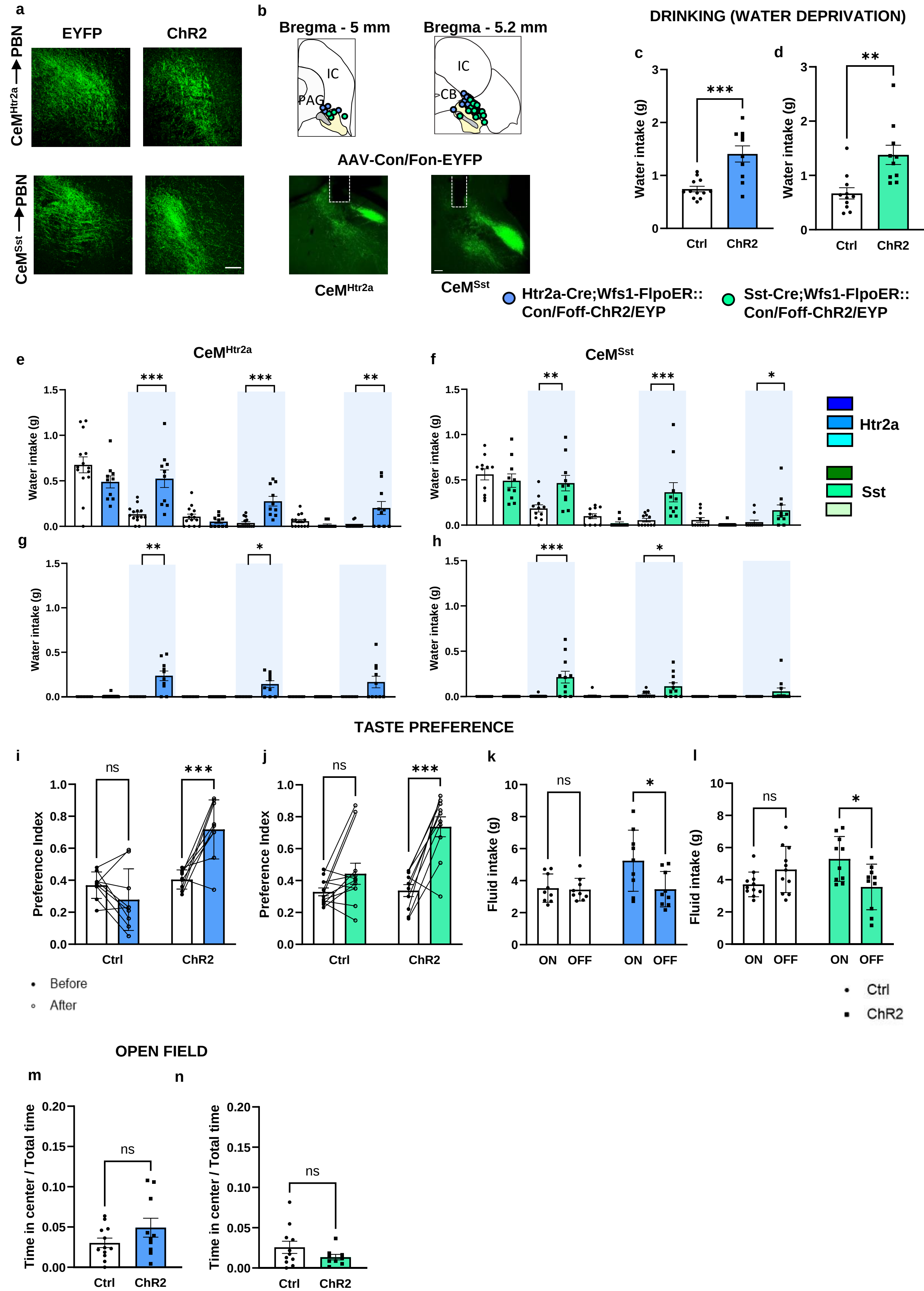

Supplementary Fig.10

Supplementary Figure 10 related to Figure 9. CeM projections to PBN drive appetitive behaviors

a. Representative images displaying the viral expression of ChR2 and EYFP of CeM^{Htr2a} and CeM^{Sst} projections to PBN. All mice used for behavior showed similar results. Scale bar: 115 µm.

b. Diagram illustrating optic fiber placement in the PBN of mice injected with Con/Foff viruses in the CeM (Htr2a-Cre;Wfs1-FlpoER::Con/Foff-ChR2/EYFP 22 out of 22 mice, Sst-Cre;Wfs1-FlpoER::Con/Foff-ChR2/EYFP 21 out of 21 mice) and representative pictures showing the optic fiber placement in the PBN. Scale bar: 126 µm.

c,d. Activation of CeM^{Htr2a} **(c)** and CeM^{Sst} **(d)** terminals to PBN increases water intake in water deprived animals (Htr2a: unpaired two-tailed t test p=0.0003, t=4.410, Sst: unpaired two-tailed t test p=0.0022, t=3.531). (CeM^{Htr2a}: n=12 Ctrl, n=10 ChR2. CeM^{Sst}: n=11 Ctrl, n=10 ChR2 mice).

e-f. Htr2a/Sst-Cre;Wfs1-FlpoER mice, water deprived, expressing ChR2 or EYFP in CeM neurons and photostimulated in the PBN, were exposed to water in a 10 min OFF/ON behavioral paradigm. The test comprised three cycles of 10 minutes without light followed by 10 minutes with light.

e. Activation of CeM^{Htr2a} PBN terminals significantly increased drinking behavior (10-20 min: unpaired two-tailed t test p=0.0002, t=4.544, 30-40 min: Mann-Whitney two-tailed U test p<0.0001, U=6, 50-60 min Mann-Whitney two-tailed U test p=0.0074, U=32). (CeM^{Htr2a}: n=14 Ctrl, n=10 ChR2 mice).

f. Activation of CeM^{Sst}::ChR2 terminals to the PBN, in the same 10 OFF/ON behavioral paradigm, promoted drinking (10-20 min: unpaired two-tailed t test p=0.0065, t=3.057, 30-40 min: Mann-Whitney two-tailed U test p=0.0003, U=8, 50-60 min: Mann-Whitney two-tailed U test p=0.0136, U=23). (CeM^{Sst}: n=11 Ctrl, n=10 ChR2 mice).

g-h. The same experiment was repeated with water-satiated animals and showed that while the control group did not consume much water, photostimulation of $CeM^{Htr2a/Sst}$::ChR2 projections to PBN was sufficient to promote water uptake (Htr2a. 10-20 min: two-tailed Wilcoxon signed rank test p=0.0078, 30-40 min two-tailed Wilcoxon signed rank test p=0.0156) **(g),** (Sst. 10-20 min: Mann-Whitney two-tailed U test p=0.0009, U=20, 30-40 min: Mann-Whitney two-tailed U test p=0.0492, U=36) **(h)**. (CeM^{Htr2a}: n=11 Ctrl, n=10 ChR2. CeM^{Sst}: n=12 Ctrl, n=11 ChR2 mice).

i. Optogenetic activation of CeM^{Htr2a} terminals to PBN changed the taste preference for the ChR2 expressing mice but not for the control group (main effect ChR2: Two-way ANOVA, F(1,16) =22.96, p=0.0002; Bonferroni post-hoc test p=0.0004). (CeM^{Htr2a}: n=9 Ctrl, n=9 ChR2 mice).

j. Activation of the CeM^{Sst} terminals to PBN reversed the initial preference of the mice (main effect TimexChR2: Two-way ANOVA, $F(1,19) = 10.38$, $p=0.0045$; Bonferroni post-hoc test $p<0.0001$). (CeM^{Sst}: n=11 Ctrl, n=10 ChR2 mice).

k,l. Photostimulation during training induced more consumption of flavored water during the light ON period for CeM^{Htr2a}::ChR2 (main effect ChR2: Two-way ANOVA, $F(1,16) = 6.920$, $p=0.0182$; Bonferroni post-hoc test $p=0.0383$) **(k)** and CeM^{Sst}::ChR2 (main effect LightxChR2: Two-way ANOVA, $F(1,19) = 9.846$, $p=0.0054$; Bonferroni post-hoc test $p=0.0213$) **(l)** terminals to PBN. (CeM^{Htr2a}: n=9 Ctrl, n=9 ChR2. CeM^{Sst}: n=11 Ctrl, n=10 ChR2 mice).

m,n. No effect was found on the time spent in the center-zone during an open-field test following activation of CeM^{Htr2a} **(m)** (unpaired two-tailed t test $p=0.1447$, t=1.518) or CeM^{Sst} **(n)** (unpaired two-tailed t test $p=0.1873$, t=1.371) PBN projections. (CeM^{Htr2a}: n=12 Ctrl, n=10 ChR2. CeM^{Sst}: n=11 Ctrl, n=9 ChR2 mice). Value = Mean± SEM.
